# Supplementary figures and images for: Two-Step Identification of N-, S-, R- and T-Cytoplasm Types in Onion Breeding Lines Using High-Resolution Melting (HRM)-Based Markers
Source: Int J Mol Sci. 2023 Jan 13;24(2):1605. doi: 10.3390/ijms24021605 (PMC9866120; doi:10.3390/ijms24021605)

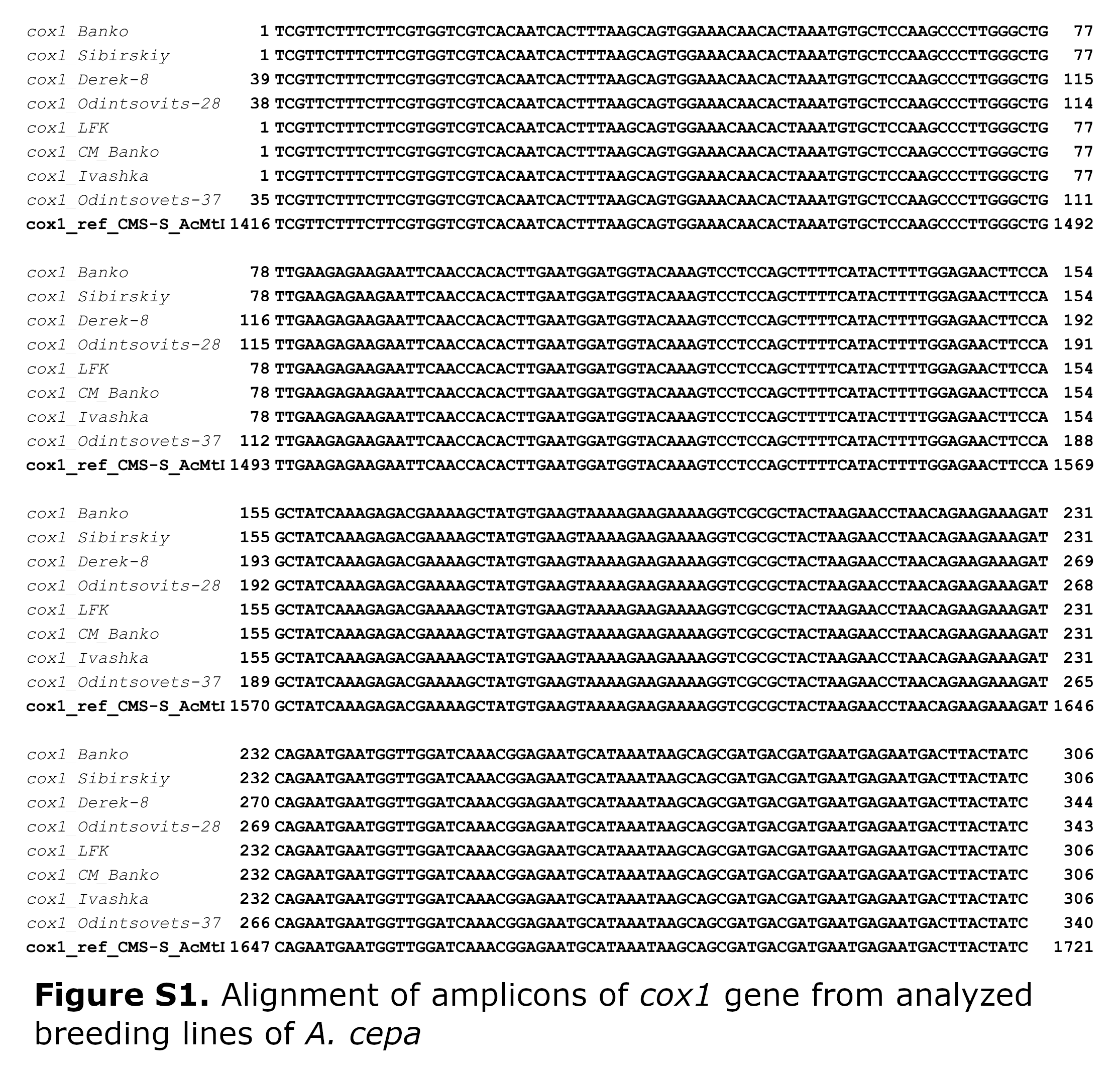

Supplement: Supplementary file 1 [file ijms-24-01605-s001.zip › Supplementary_Figure S1.tiff]

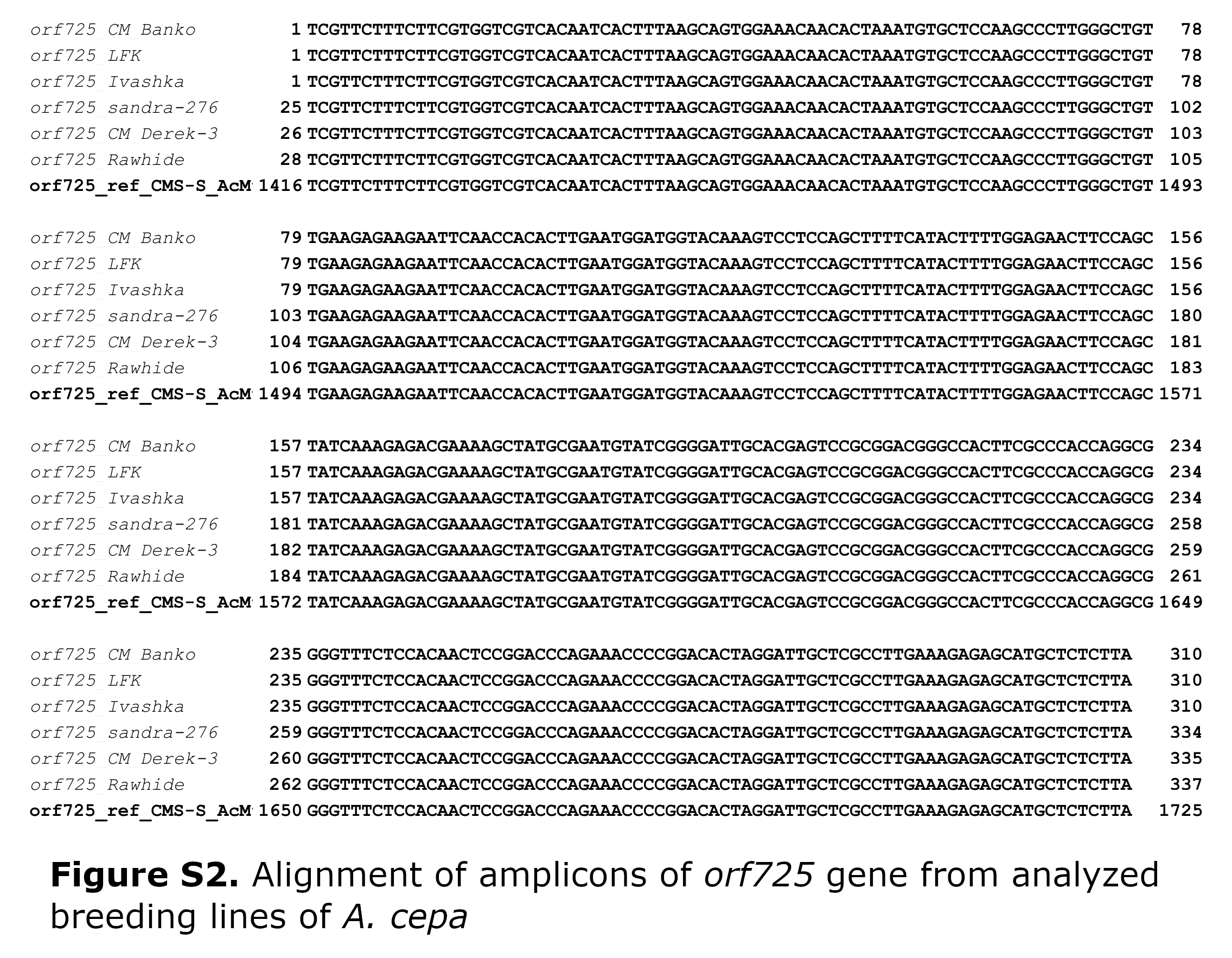

Supplement: Supplementary file 1 [file ijms-24-01605-s001.zip › Supplementary_Figure S2.tiff]
